# Triglyceride-glucose index and estimated 10-year risk of a first hard cardiovascular event

**DOI:** 10.3389/fcvm.2022.994329

**Published:** 2023-01-09

**Authors:** Hua Qu, Lin-zi Long, Li Chen, Han-tao Wu, Chang-geng Fu, Shan-shan Zhang

**Affiliations:** ^1^Xiyuan Hospital, China Academy of Chinese Medical Sciences, Beijing, China; ^2^National Clinical Research Center for Chinese Medicine Cardiology, Beijing, China; ^3^NMPA Key Laboratory for Clinical Research and Evaluation of Traditional Chinese Medicine, Beijing, China; ^4^Beijing Liaoweiyuan Institute of Traditional Chinese Medicine, Beijing, China; ^5^Xibeiwang Town Community Health Service Center, Beijing, China

**Keywords:** TyG index, 10-year risk, atherosclerotic cardiovascular disease, NHANES, cross-sectional study

## Abstract

**Background:**

Whether Triglyceride-glucose (TyG) index is associated with 10-year risk of a first hard atherosclerotic cardiovascular disease (ASCVD) event in the United States remains unclear.

**Methods:**

In this cross-sectional study, the participants, ranged from 40 to 79 years old, were from the National Health and Nutrition Examination Survey (NHANES) between 1999 and 2018. TyG index was the independent variable and 10-year risk of a first hard ASCVD was the dependent variable. The other variables, such as age, gender, race, body mass index (BMI), hypertension treatment states, smoking states and low-density lipoprotein cholesterol (LDL-C) et al. were considered as the potential confounding factors. Multivariate linear regression models and smooth curve fittings were used to evaluate the association between TyG index and 10-year risk of a first hard ASCVD event.

**Results:**

A total of 2,142 participants were included in the analysis. The results showed that TyG index was associated with an increased 10-year risk of a first hard ASCVD event [β = 2.208, 95% (1.716, 2.700), *P* < 0.00001]. The association had statistical significance in both men [β = 3.862 95% CI (3.274, 4.450), *P* < 0.00001] and women [β = 1.067, 95% CI (0.286, 1.849), *P* = 0.00756)] according to subgroup analysis. Smooth curve fittings revealed that TyG index was linearly associated with 10-year risk of ASCVD in both male and female.

**Conclusion:**

Triglyceride-glucose index was associated with an increased 10-year risk of a first hard ASCVD event in the United States, suggesting it is necessary to monitor and control an appropriate range of TyG index.

## Introduction

Cardiovascular disease (CVD) is the leading cause of morbidity and mortality all over the world (1). Despite the clinical application of anti-platelet agents and statins, the patients with CVD remain high risk of recurrent adverse cardiovascular events. Therefore, identifying the early indicator of CVD is significant, and will be beneficial for instructing further healthcare (2, 3).

The homeostasis model assessment estimated insulin resistance (HOMA-IR) index is used to evaluate β-cell function and insulin resistance (IR), but the effectiveness of HOMA-IR is influenced by insulin treatment (4). The triglyceride-glucose (TyG) index, calculated by Ln[fasting blood TG (mg/dL) × fasting glucose (mg/dL)/2)], has been confirmed more superior than HOMA-IR in assessing IR in individuals with or without diabetes (5). Recently, TyG index was also considered as an indicator of CVD or cardiovascular risk factors. Park et al.’s study showed that increased TyG index was associated with progression of carotid arterial stenosis, and Li et al. revealed that TyG index was positive related with the incidence of carotid artery plaques in patients with coronary heart disease (6, 7). Moreover, Sang et al. demonstrated that TyG index was independently associated with increased arterial stiffness (8). A multicenter retrospective study, including 731 patients with coronary heart disease, showed that TyG index influenced the severity of CAD, and increased TyG index could predict a high risk of multi-vessel lesions. Many clinical studies also showed that TyG index might be used as a hallmark of cardiovascular events in CVD patients (7, 9–13).

In addition, Ding et al. performed a meta-analysis of cohort studies which included more than 5 million participants, and the results demonstrated that compared with the lowest TyG index, the highest TyG index were independently associated with a higher incidence of atherosclerotic cardiovascular disease (ASCVD) in the participants without CVD at baseline (10). However, the included studies were performed in Asian or European, whether TyG index is associated with cardiovascular risk in the United States remains unclear.

Here, we conducted a cross-sectional study to estimate the association between TyG index and 10-year risk of a first hard ASCVD event (defined as non-fatal myocardial infarction or coronary heart disease death or stroke, over a 10-year period among people free from ASCVD at the beginning of the period) according to the 2013 ACC/AHA guideline on the Assessment of Cardiovascular Risk (14) using a large-scale database from National Health and Nutrition Examination Survey (NHANES).

## Materials and methods

### Study population

National Health and Nutrition Examination Survey is a population-based national survey that collected information regarding health and nutrition status in the United States with biennial cycles.^1^ Our analysis collected the data of 10 cycles between 1999 and 2018. The risk of a first hard ASCVD event in adults aged from 40 to 79 years without diagnosed ASCVD was estimated according to the 2013 ACC/AHA guideline (14). The participants were included if they met following criteria: (1) 40 to 79 years old, (2) without diagnosed ASCVD, (3) the level of high-density lipoprotein cholesterol (HDL-C) ranged from 20 mg/dl to 100 mg/dl, (4) the level of total cholesterol (TC) ranged from 130 to 320 mg/dl, and (5) the systolic blood pressure (SBP) ranged from 90 to 200 mmHg.

Finally, 2142 participants were included in the analyses after excluding the participants missing TyG data (*n* = 3136). The ethics review board of the National Center for Health Statistics approved all NHANES protocols and informed consents were obtained from all participants (15).

### Variables

Continuous variables included age, body mass index (BMI), SBP, diastolic blood pressure (DBP), TC, TG, HDL-C, low density lipoprotein cholesterol (LDL-C), TyG index. Categorical variables included gender, race, smoking status, hypertension treatment status and diabetes status. The 10-year risk of a first hard ASCVD event was calculated according to the 2013 ACC/AHA Guideline on the Assessment of Cardiovascular Risk, which can be accessed by https://tools.acc.org/ASCVD-Risk-Estimator-Plus/#!/calculate/estimate/ (14). The TyG index was calculated by Ln [fasting TG (mg/dL) × fasting glucose (mg/dL)/2)].

### Statistical analysis

Continuous variables were represented as mean and standard deviation (SD) values if the data was normally distributed, or as median values and interquartile ranges otherwise; comparisons between groups were analyzed with Student’s *t* test or one-way analysis of variance. Categorical variables were described as percentages and compared by χ^2^ testing. We performed multicollinearity between variables, and the covariates were included as potential confounders in the final models if they changed the estimates of TyG index on 10-year risk of a first hard cardiovascular event by more than 10% or were significantly associated with 10-year risk of a first hard cardiovascular event (*P* < 0.01). Multivariable linear regression was used to assess the association between TyG index and 10-year risk of ASCVD event, and shown as model 1 (unadjusted), model 2 (adjusted for age, gender and race), model 2 (adjusted for m age, gender, race, BMI, SBP, DBP, hypertension treatment, smoking, diabetes, LDL-C, and fasting glucose). All statistical analyses were performed with R (The R Foundation; version 3.4.3)^2^ and EmpowerStats software (X&Y Solutions, Inc., Boston, MA, USA).^3^

## Results

### Characteristics of included participants

A total of 2,142 participants were included in our analyses, the characteristics of participants are presented in Table 1. The participants were classified based on TyG index quartiles (Q1, 7.069–8.448; Q2, 8.448–8.866; Q3, 8.866–9.318; Q4, 9.318–12.309). There were obvious differences in age, gender, race, BMI, lipid profiles, fasting glucose, SBP, DBP, and diabetes status among four groups (*P* < 0.05). And smoking status and hypertension treatment status are comparable among four groups (*P* > 0.05).

**TABLE 1:** Baseline characteristic of participants.

TyG index	Q1 (7.069–8.448)	Q2 (8.448–8.866)	Q3 (8.866–9.318)	Q4 (9.318–12.309)	*P*-value
*N*	536	535	535	536	
Age	60.63 ± 10.12	60.16 ± 10.51	61.35 ± 9.69	58.82 ± 10.32	<0.001
Gender					0.013
Men	281 (52.43%)	284 (53.08%)	297 (55.51%)	329 (61.38%)	
Women	255 (47.57%)	251 (46.92%)	238 (44.49%)	207 (38.62%)	
Race					<0.001
Non-Hispanic black	214 (39.93%)	144 (26.92%)	93 (17.38%)	73 (13.62%)	
Other	322 (60.07%)	391 (73.08%)	442 (82.62%)	463 (86.38%)	
BMI	28.84 ± 6.93	30.39 ± 6.70	31.48 ± 6.54	31.92 ± 5.66	<0.001
**Lipid profile**					
TC (mg/dl)	189.55 ± 32.75	197.09 ± 35.18	200.11 ± 36.46	208.28 ± 39.62	<0.001
HDL-C (mg/dl)	62.01 ± 14.83	55.04 ± 13.66	48.97 ± 11.62	42.58 ± 11.38	<0.001
TG (mg/dl)	71.92 ± 17.31	110.44 ± 19.63	153.19 ± 33.20	279.04 ± 155.12	<0.001
LDL-C (mg/dl)	113.21 ± 30.25	120.14 ± 32.21	120.46 ± 33.62	114.55 ± 34.89	<0.001
Fasting glucose (mg/dl)	99.39 ± 14.82	106.54 ± 17.23	118.99 ± 29.12	148.83 ± 65.06	<0.001
SBP	132.60 ± 18.41	131.43 ± 18.76	134.32 ± 19.80	134.33 ± 19.46	0.032
DBP	71.37 ± 14.02	71.88 ± 13.80	72.63 ± 13.37	73.99 ± 14.32	0.013
Hypertension treatment					0.122
Yes	455 (84.89%)	452 (84.49%)	472 (88.22%)	473 (88.25%)	
No	81 (15.11%)	83 (15.51%)	63 (11.78%)	63 (11.75%)	
Diabetes					<0.001
Yes	63 (11.75%)	70 (13.08%)	152 (28.41%)	222 (41.42%)	
No	473 (88.25%)	465 (86.92%)	383 (71.59%)	314 (58.58%)	
Smoker					0.414
Yes	178 (33.21%)	197 (36.82%)	184 (34.39%)	201 (37.50%)	
No	358 (66.79%)	338 (63.18%)	351 (65.61%)	335 (62.50%)	
10-year risk	14.43 ± 11.66	14.64 ± 12.14	18.26 ± 13.51	19.75 ± 14.43	<0.001

Mean ± SD for continuous variables. (%) for categorical variables. TyG, triglyceride-glucose; BMI, body mass index; SBP, systolic blood pressure; DBP, diastolic blood pressure; TC, total cholesterol; HDL-C, high-density lipoprotein cholesterol; TG, triglyceride; LDL-C, low density lipoprotein cholesterol.

### TyG index and 10-year risk of a first hard ASCVD event

**FIGURE 1:**
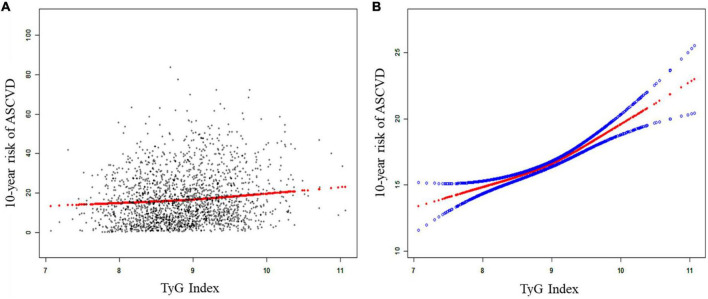
The association between TyG index and the 10-year risk of a first hard ASCVD event. **(A)** The linear association between TyG index and the 10-year risk of a first hard ASCVD event. Black points represent samples, the red line represent the fitting line. **(B)** The smooth curve between TyG index and the 10-year risk of a first hard ASCVD event. Solid red line represents the smooth curve. Blue lines represent the 95% of confidence interval. Age, gender, race, body mass index, systolic blood pressure, diastolic blood pressure, hypertension treatment status, smoking status, diabetes status, and low-density lipoprotein cholesterol were adjusted. ASCVD, atherosclerotic cardiovascular disease; TyG, triglyceride-glucose.

The results from regression analyses were described in Table 2. We adjusted no confounding factor in model 1; adjusted age, gender and race in model 2; and adjusted age, gender, race, BMI, SBP, DBP, hypertension treatment status, smoking status, diabetes and LDL-C in model 3. The results demonstrated that TyG index was associated with an increased 10-year risk of a first hard ASCVD event in model 1 [β = 3.680, 95% CI (2.847, 4.513)], model 2 [β = 4.950, 95% CI (4.393, 5.508)] or model 3[β = 2.208, 95% (1.716, 2.700)]. Compared with the TyG index in Q1 (the lowest quartile), the 10-year risk of a first hard ASCVD event increased in Q3 quartile [(β = 1.177, 95% CI (0.493, 1.860)], and in Q4 quartile [(β = 3.151, 95% CI (2.382, 3.920)]. We performed generalized additive models and smooth curve fittings to evaluate the associations between TyG index and the 10-year risk (Figure 1). There was a linear relationship between TyG index and the 10-year risk, which indicated that the 10-year risk of a first hard ASCVD event increased with TyG index.

**TABLE 2:** Linear regression analyses of TyG index and 10-year risk of a first hard ASCVD event.

	Model 1	Model 2	Model 3
	β (95% CI), *P*-value	β (95% CI), *P*-value	β (95% CI), *P*-value
TyG index	3.680 (2.847, 4.513), <0.00001	4.950 (4.393, 5.508), <0.00001	2.208 (1.716, 2.700) <0.00001
**TyG index (quartiles)**			
Q2 vs. Q1	0.214 (−1.341, 1.769), 0.78734	1.244 (0.213, 2.275), 0.01812	0.673 (0.021, 1.325) 0.04304
Q3 vs. Q1	3.830 (2.275, 5.385), <0.00001	4.138 (3.094, 5.181), <0.00001	1.177 (0.493, 1.860) 0.00075
Q4 vs. Q1	5.328 (3.774, 6.882), <0.00001	7.643 (6.590, 8.696), <0.00001	3.151 (2.382, 3.920) <0.00001

Model 1 adjust for none; Model 2 adjust for age, gender and race; model 3 adjust for age, gender, race, body mass index, systolic blood pressure, diastolic blood pressure, hypertension treatment, smoking, diabetes, low density lipoprotein cholesterol and fasting glucose. TyG, triglyceride-glucose; CI, confidence interval.

### Stratified analysis based on gender between TyG index and the 10-year risk of a first hard ASCVD event

**FIGURE 2:**
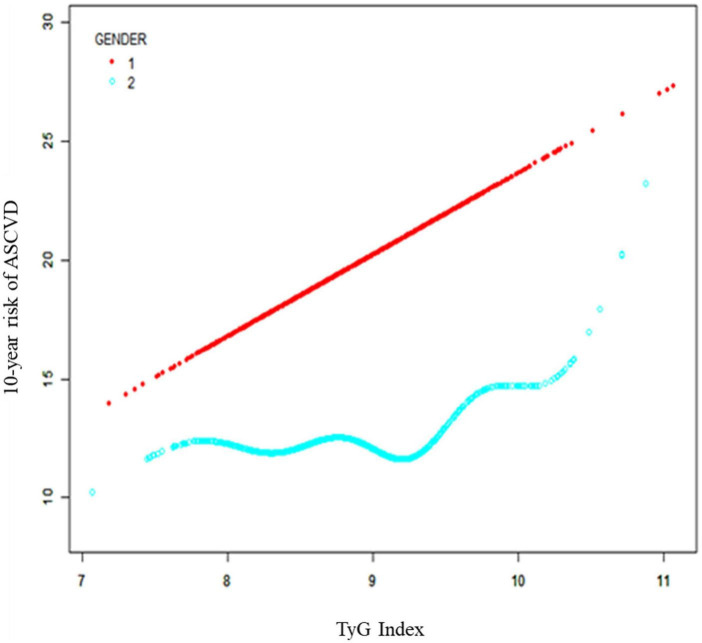
Subgroup analysis stratified by gender between TyG index and 10-year risk of a first hard ASCVD event. Age, race, body mass index, systolic blood pressure, diastolic blood pressure, hypertension treatment, smoking, diabetes, and low-density lipoprotein cholesterol were adjusted. ASCVD, atherosclerotic cardiovascular disease; TyG, triglyceride-glucose.

Subgroup analysis stratified by gender was performed, and significant associations between TyG index and 10-year risk of a first hard ASCVD event were observed in both men [β = 3.862, 95% CI (3.274, 4.450), *P* < 0.00001] and women [β = 1.067, 95% CI (0.286, 1.849), *P* = 0.00756)] (Table 3). Smooth curve fittings and generalized additive models were used to characterize the association between TyG index and the 10-year risk of a first hard ASCVD event (Figure 2).

**TABLE 3:** Subgroup analysis stratified by gender between TyG index and 10-year risk of a first hard ASCVD event.

	Model 1	Model 2	Model 3
	β (95% CI), *P*-value	β (95% CI), *P*-value	β (95% CI), *P*-value
Male	2.917 (1.791, 4.042), <0.00001	6.117 (5.360, 6.873), <0.00001	3.862 (3.274, 4.450) <0.00001
Female	3.894 (2.766, 5.021), <0.00001	3.825 (3.010, 4.641), <0.00001	1.067 (0.286, 1.849) 0.00756

Model 1 adjust for none; Model 2 adjust for age, gender and race; model 3 adjust for age, gender, race, body mass index, systolic blood pressure, diastolic blood pressure, hypertension treatment, smoking, diabetes, low density lipoprotein cholesterol. CI, confidence interval.

## Discussion

In the present study, 2,142 participants aged from 40 to 79 were included in the analyses to evaluate the associations between TyG index and the 10-year risk of a first hard ASCVD event. As we known, this is the first study to reveal the positive association between TyG index and 10-year risk of a first hard ASCVD event in the United States. In addition, the subgroup analysis based on gender showed that TyG index was associated with the 10-year risk of a first hard ASCVD event in both male and female. And we also identified the threshold effect of TyG index on ASCVD events in male was 9.812.

Despite significant advances in the prevention and treatment of ASCVDs, ASCVDs remain one of the leading causes of death worldwide (16, 17). Established risk factors of ASCVDs include age, the male sex, family history of ASCVDs, obesity, hypertension, hypercholesteremia, and diabetes (18, 19). However, recent studies also demonstrated that some patients without these risk factors may still develop ASCVDs, thus highlighting the importance of identifying novel risk factors for ASCVDs in the general population (20–22). TyG index has been identified as an indicator of cardiovascular events in Asia or Europe (10, 23), however, whether TyG index could be a hallmark of CVD incidence in the United States remains unclear because of the differences in the genes of CVD susceptibility and risk factors (24, 25). In the present study, we used the data from NHANES, which included nationally representative samples in the United States. And the results demonstrated that there is a positive association between TyG index and the 10-year risk of a first hard ASCVD event in the United States regardless of confounders, such as age, gender, race, BMI, SBP, DBP, hypertension treatment status, smoking status, diabetes status, and LDL-C. Additionally, even though significant associations between TyG index and 10-year risk of a first hard ASCVD event were observed in both men and women, the risk of ASCVD events increased more in male than those in female as each unit of TyG index increased (3.862 vs. 1.067%), indicating that TyG index might be more relevant to incidence of CVD in male than female. These difference between male and female may be due to the combinations of other risk factors (26) and these exposures could substantially increase 10-year risk of a first hard ASCVD event and enlarged the potentially effect of TyG index (27).

A strength of this study is that we firstly explored the association between TyG index and 10-year risk of a first hard ASCVD event in the United States. There were several limitations in our study. Firstly, we could not obtain the total covariates, such as atherosclerotic history, because some data were not collected in the survey. Secondly, races were divided into non-Hispanic Black people and others to calculate the 10-year risk of a first hard cardiovascular event, so other categories cannot be calculated. Finally, the present analysis was based on cross-sectional study, the specific causality relationship between TyG index and 10-year risk of a first hard cardiovascular event in the United States should be validated in prospective studies.

## Conclusion

Triglyceride-glucose index was associated with an increased 10-year risk of a first hard ASCVD event in the United States using a cross-sectional database, suggesting it is necessary to monitor and control an appropriate range of TyG index.

## Data availability statement

The original contributions presented in this study are included in the article/supplementary material, further inquiries can be directed to the corresponding authors.

## Ethics statement

The studies involving human participants were reviewed and approved by the National Center for Health Statistics. The patients/participants provided their written informed consent to participate in this study.

## Author contributions

HQ and L-ZL: writing the manuscript and data analysis. LC: revising the manuscript. H-TW: statistics and analyses. C-GF and S-SZ: revising and editing. All authors contributed to the article and approved the submitted version.

## Footnotes

1
www.cdc.gov/nchs/nhanes/

2
http://www.R-project.org

3
http://www.empowerstats.com
